# Effect of Ivabradine on a Hypertensive Heart and the Renin-Angiotensin-Aldosterone System in *L*-NAME-Induced Hypertension

**DOI:** 10.3390/ijms19103017

**Published:** 2018-10-03

**Authors:** Fedor Simko, Tomas Baka, Marko Poglitsch, Kristina Repova, Silvia Aziriova, Kristina Krajcirovicova, Stefan Zorad, Michaela Adamcova, Ludovit Paulis

**Affiliations:** 1 Institute of Pathophysiology, Faculty of Medicine, Comenius University, Sasinkova 4, 81108 Bratislava, Slovakia; tomasko.baka@gmail.com (T.B.); repova.k@gmail.com (K.R.); silvia.aziriova@gmail.com (S.A.); krikratina@gmail.com (K.K.); ludovit.paulis@gmail.com (L.P.); 2 3rd Department of Internal Medicine, Faculty of Medicine, Comenius University, 83305 Bratislava, Slovakia; 3 Institute of Experimental Endocrinology, Biomedical Research Center, Slovak Academy of Sciences, 84505 Bratislava, Slovakia; stefan.zorad@savba.sk; 4 Attoquant Diagnostics, 1030 Vienna, Austria; marko.poglitsch@attoquant.com; 5 Department of Physiology, School of Medicine, Charles University, 50003 Hradec Kralove, Czech Republic; adamcova@lfhk.cuni.cz; 6 Institute of Normal and Pathological Physiology, Center for Experimental Medicine, Slovak Academy of Sciences, 81371 Bratislava, Slovakia

**Keywords:** ivabradine, *L*-NAME, hypertension, fibrosis, angiotensin II, aldosterone, heart function

## Abstract

Ivabradine, the selective inhibitor of the If current in the sinoatrial node, exerts cardiovascular protection by its bradycardic effect and potentially pleiotropic actions. However, there is a shortage of data regarding ivabradine’s interaction with the renin-angiotensin-aldosterone system (RAAS). This study investigated whether ivabradine is able to protect a hypertensive heart in the model of *L*-NAME-induced hypertension and to interfere with the RAAS. Four groups (*n* = 10/group) of adult male Wistar rats were treated as follows for four weeks: control, ivabradine (10 mg/kg/day), *L*-NAME (40 mg/kg/day), and *L*-NAME plus ivabradine. *L*-NAME administration increased systolic blood pressure (SBP) and left ventricular (LV) weight, enhanced hydroxyproline concentration in the LV, and deteriorated the systolic and diastolic LV function. Ivabradine reduced heart rate (HR) and SBP, and improved the LV function. The serum concentrations of angiotensin Ang 1–8 (Ang II), Ang 1–5, Ang 1–7, Ang 1–10, Ang 2–8, and Ang 3–8 were decreased in the *L*-NAME group and ivabradine did not modify them. The serum concentration of aldosterone and the aldosterone/Ang II ratio were enhanced by *L*-NAME and ivabradine reduced these changes. We conclude that ivabradine improved the LV function of the hypertensive heart in *L*-NAME-induced hypertension. The protective effect of ivabradine might have been associated with the reduction of the aldosterone level.

## 1. Introduction

Ivabradine is a novel selective inhibitor of hyperpolarization-activated cyclic nucleotide-gated channels, inducing heart rate (HR) reduction [1]. After a decade of disappointing trials, ivabradine has created new hope for heart failure (HF) patients. In the SHIFT study, ivabradine reduced the composite endpoint of cardiovascular death and hospital admission for the deterioration of HF in symptomatic patients with systolic dysfunction and sinus rhythm with an HR equal to or higher than 70 beats per minute, despite the treatment with beta-blockers [2]. The bradycardic effect is considered to be the principal pathophysiological mechanism of ivabradine’s benefit [2]. Moreover, a number of pleiotropic actions of ivabradine have been described during recent years [3]. Ivabradine was shown to attenuate chemokine-induced migration of CD4-positive lymphocytes in vivo [4], reduce vascular nicotinamide adenine dinucleotide phosphate (NADPH) oxidase activity in apolipoprotein E (ApoE)-deficient mice [5], improve the functioning of myocardial mitochondria in an infarcted pig heart [3], and reduce high-sensitivity C-reactive protein levels in patients with acute coronary syndrome [6]. As a result of these anti-inflammatory, antiapoptotic, and oxidative stress reducing actions, ivabradine improved the endothelial function in ApoE knockout mice [7]; attenuated systolic and diastolic dysfunction, left ventricular hypertrophy (LVH), and fibrosis in angiotensin II-induced HF in rats [8]; and exerted an antihypertrophic effect on the aorta in spontaneously hypertensive rats [9]. However, data regarding the potential protection of ivabradine on a hypertensive heart are sparse.

The aim of our study was to show whether ivabradine is able to protect a hemodynamically overloaded heart in the model of N^G^-nitro-*L*-arginine methyl ester (*L*-NAME)-induced hypertension. Moreover, since heart remodeling is associated with the activation of angiotensin II or aldosterone, we investigated whether the potential effects of ivabradine in *L*-NAME-hypertension are related to its interference with the renin-angiotensin-aldosterone system (RAAS). 

## 2. Results

### 2.1. Cardiovascular Parameters

**Figure 1:**
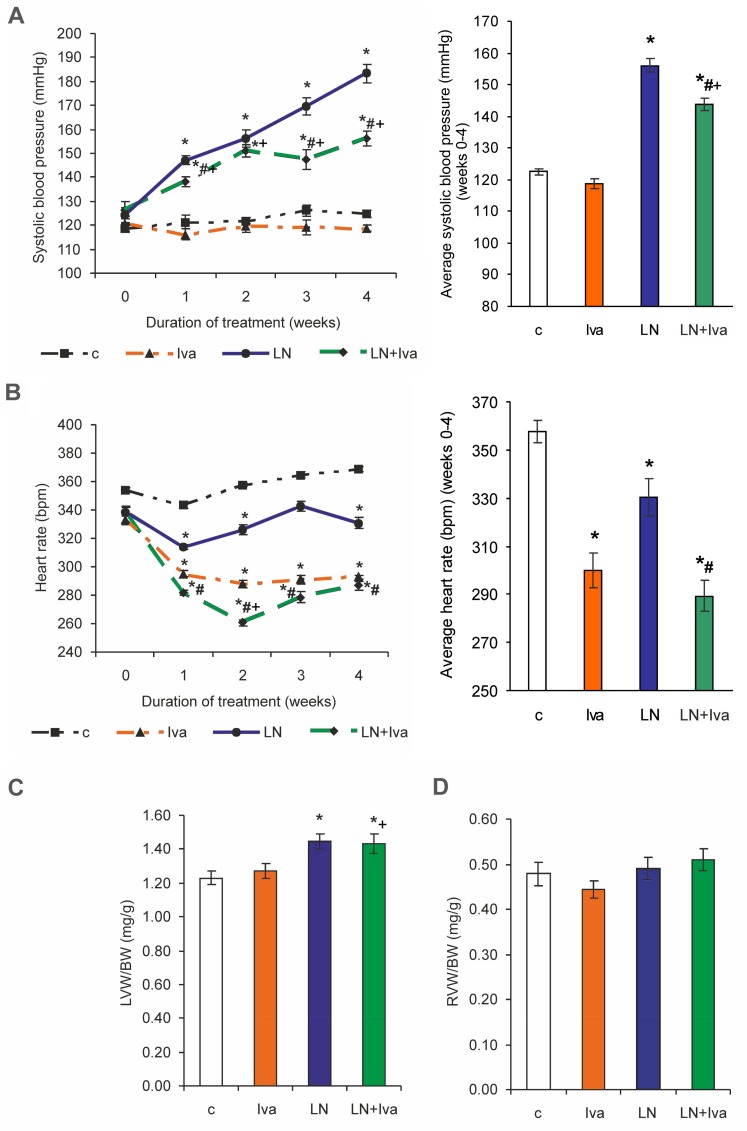
The influence of ivabradine (LN + Iva) on systolic blood pressure (SBP) and average SBP (**A**), heart rate (HR) and average HR (**B**), relative left ventricular weight (LVW/BW) (**C**), and relative right ventricular weight (RVW/BW) (**D**) in L-NAME-treated rats. Wistar controls (c); L-NAME (LN); ivabradine (Iva); n = 10 per group; * *p* < 0.05 vs. c; # *p* < 0.05 vs. LN; + *p* < 0.05 vs. Iva; results are expressed as mean ± SEM.

Systolic blood pressure (SBP) was 124.68 ± 1.52 mmHg in the control and was enhanced to 183.40 ± 3.82 mmHg in the L-NAME group (by 47%, *p* < 0.05) after four weeks of treatment. SBP decreased by ivabradine treatment compared to the L-NAME group (by 15%, *p* < 0.05) (Figure 1A). Average SBP was 122.54 ± 0.98 mmHg in the control and was enhanced to 156.09 ± 2.29 mmHg in the L-NAME group (by 27%, *p* < 0.05) after four weeks of treatment. Average SBP decreased by ivabradine treatment compared to the L-NAME group (by 8%, *p* < 0.05) (Figure 1A). HR was 368.63 ± 4.97 bpm in the control and was reduced to 330.83 ± 9.12 bpm in the L-NAME group (by 10%, *p* < 0.05). Ivabradine significantly decreased HR in both the control and L-NAME groups (by 21% and 13%, respectively, *p* < 0.05 for both) (Figure 1B). The average HR was 357.65 ± 4.49 bpm in the control and was reduced to 330.38 ± 7.56 bpm in the L-NAME group (by 8%, *p* < 0.05). Ivabradine decreased the average HR in both the control and L-NAME groups (by 16% and 12%, respectively, *p* < 0.05 for both) (Figure 1B). The left ventricular weight (LVW)/body weight (BW) ratio was 1.23 ± 0.04 mg/g in the control and was enhanced to 1.45 ± 0.04 mg/g in the L-NAME group (by 18%, *p* < 0.05). Ivabradine had no effect on the LVW/BW ratio (Figure 1C). The right ventricular weight (RVW)/BW ratio was 0.48 ± 0.03 mg/g and 0.49 ± 0.03 mg/g, in the control and L-NAME groups, respectively (non-significant—ns), and ivabradine had no effect (Figure 1D).

### 2.2. Hydroxyproline Concentration and Content in the Soluble and Insoluble Collagen and Total Hydroxyproline

**Figure 2:**
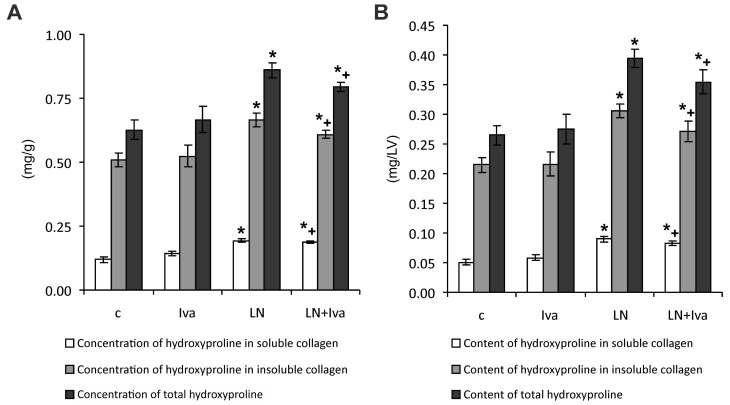
The influence of ivabradine (LN + Iva) on the hydroxyproline concentration in soluble and insoluble collagenous proteins and on the total hydroxyproline concentration (**A**) and content (**B**) in the left ventricle of *L*-NAME-treated rats. Wistar controls (c); *L*-NAME (LN); ivabradine (Iva); *n* = 10 per group; * *p* < 0.05 vs. c; + *p* < 0.05 vs. Iva; results are expressed as mean ± SEM.

After four weeks of treatment, the hydroxyproline concentration in the soluble collagenous protein was 0.119 ± 0.011 mg/g in the control and was enhanced to 0.194 ± 0.007 mg/g in the *L*-NAME group (by 63%, *p* < 0.05). Ivabradine had no effect on the hydroxyproline concentration in soluble collagen (Figure 2A).

The hydroxyproline concentration in the insoluble collagen was 0.507 ± 0.027 mg/g in the control and was enhanced to 0.665 ± 0.026 mg/g in the *L*-NAME group (by 31%, *p* < 0.05). Ivabradine had no effect on the hydroxyproline concentration in insoluble collagen of hypertensive rats (Figure 2A).

The total hydroxyproline concentration was 0.626 ± 0.038 mg/g in the control and was enhanced to 0.860 ± 0.030 mg/g in the *L*-NAME group (by 37%, *p* < 0.05). Ivabradine had no effect on the total hydroxyproline concentration (Figure 2A).

The hydroxyproline content in the soluble collagenous protein was 0.050 ± 0.005 mg/LV in the control and was enhanced to 0.090 ± 0.0041 mg/LV in the *L*-NAME group (by 80%, *p* < 0.05). Ivabradine had no effect on the hydroxyproline content in the soluble collagen (Figure 2B).

The hydroxyproline content in the insoluble collagen was 0.215 ± 0.012 mg/LV in the control and was enhanced to 0.305 ± 0.012 mg/LV in the *L*-NAME group (by 42%, *p* < 0.05). Ivabradine had no effect on the hydroxyproline content in the insoluble collagen of hypertensive rats (Figure 2B).

The total hydroxyproline content was 0.265 ± 0.017 mg/LV in the control and was enhanced to 0.395 ± 0.015 mg/LV in the L-NAME group (by 49%, *p* < 0.05). Ivabradine had no effect on the total hydroxyproline content (Figure 2B).

### 2.3. The Serum Concentration of Angiotensins and Aldosterone, Concentration of Angiotensin in the LV and Renin Activity (Measured and Surrogate)

**Figure 3:**
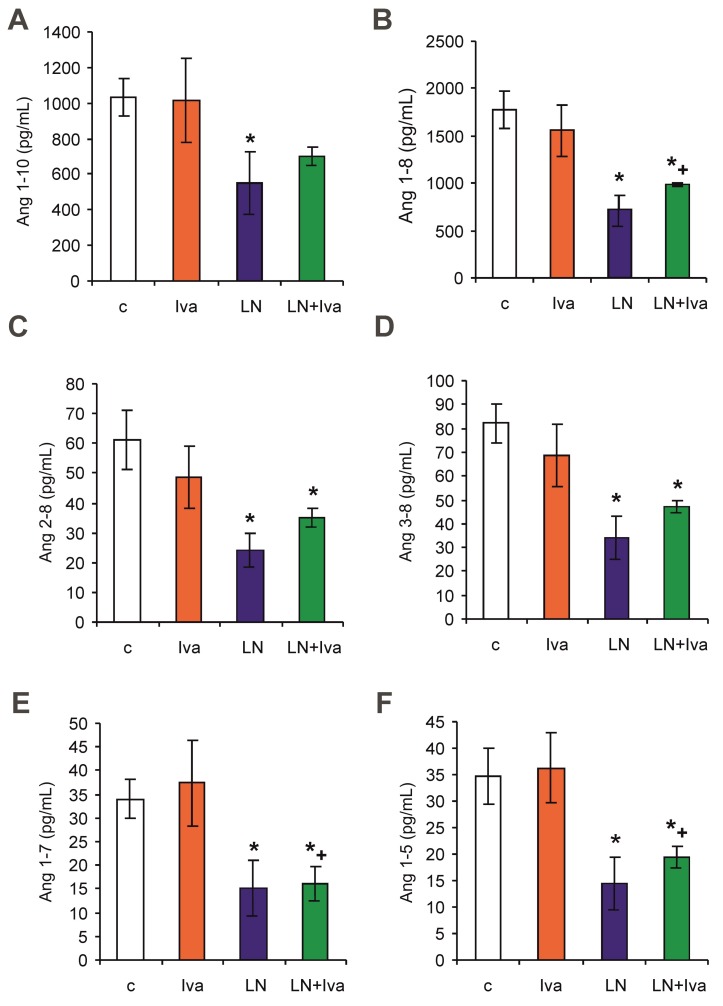

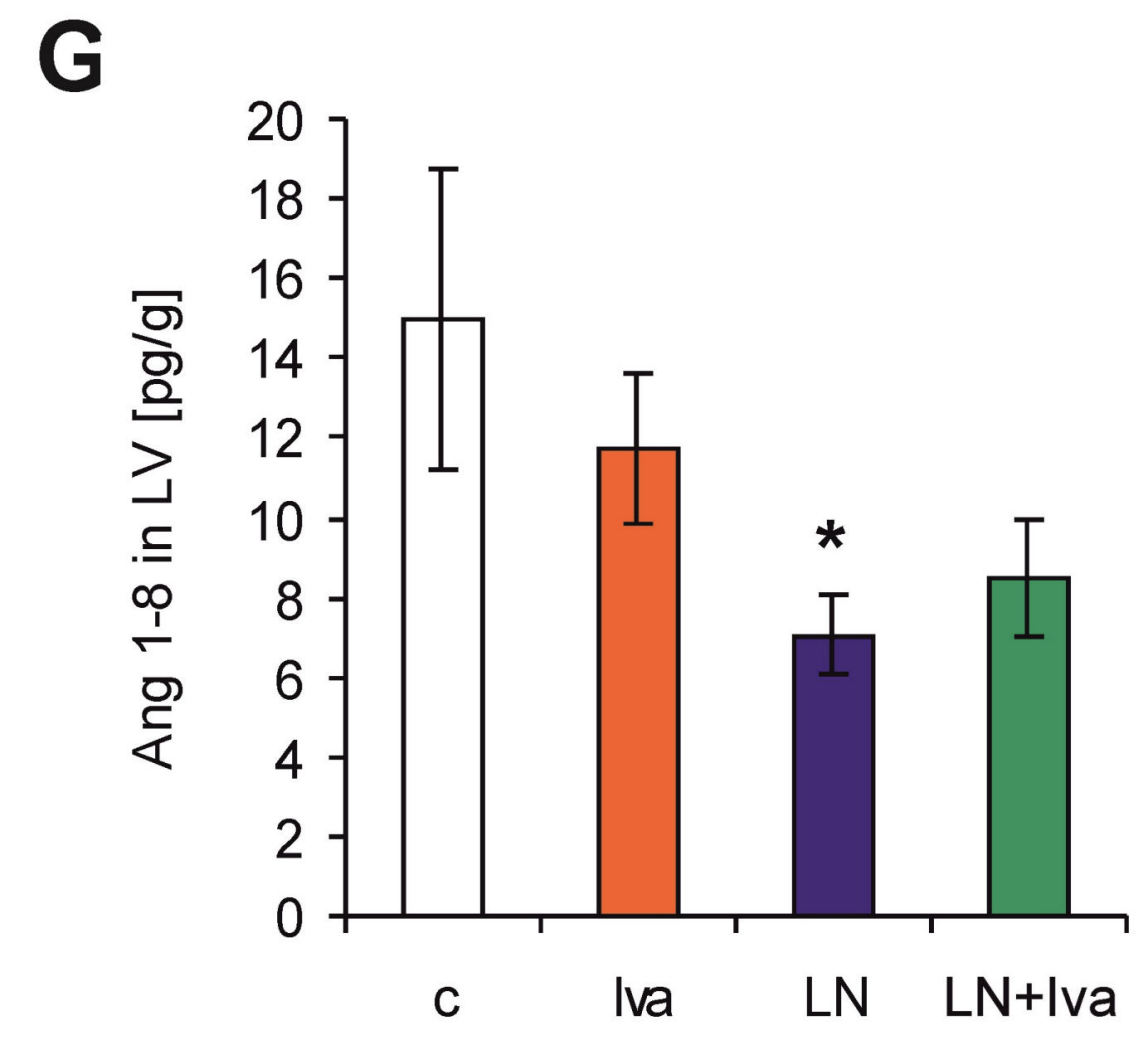
The influence of ivabradine (LN + Iva) on the serum level of angiotensin 1–10 (Ang I) (**A**), angiotensin 1–8 (Ang II) (**B**), angiotensin 2–8 (Ang 2–8) (**C**), angiotensin 3–8 (Ang 3–8) (**D**), angiotensin 1–7 (Ang 1–7) (**E**), angiotensin 1–5 (Ang 1–5) (**F**), and Ang II in the tissue of the left ventricle (LV) (**G**) in *L*-NAME-treated rats. Wistar controls (c); *L*-NAME (LN); ivabradine (Iva); *n* = 6 per group; * *p* < 0.05 vs. c; + *p* < 0.05 vs. Iva; results are expressed as mean ± SEM.

After four weeks of treatment, the serum equilibrium level of Ang 1–8 was 1773.46 ± 192.61 pg/mL in the control group and was reduced by *L*-NAME to 717.23 ± 158.90 pg/mL (60%, *p* < 0.05) (Figure 3B); the level of Ang 1–7 was 33.93 ± 4.15 pg/mL in the control group and was reduced by *L*-NAME to 15.17 ± 5.73 pg/mL (55%, *p* < 0.05) (Figure 3E); the level of Ang 1–5 was 34.61 ± 5.33 pg/mL in the control group and was reduced by *L*-NAME to 14.49 ± 4.78 pg/mL (58%, *p* < 0.05) (Figure 3F); the level of Ang 1–10 was 1033.49 ± 106.13 pg/mL in the control group and was reduced by *L*-NAME to 548.55 ± 175.43 pg/mL (47%, *p* < 0.05) (Figure 3A); the level of Ang 2–8 was 61.28 ± 9.88 pg/mL in the control group and was reduced by *L*-NAME to 23.85 ± 5.99 pg/mL (61%, *p* < 0.05) (Figure 3C); and the level of Ang 3–8 was 82.25 ± 8.13 pg/mL in the control group and was reduced by *L*-NAME to 34.17 ± 9.12 pg/mL (58%, *p* < 0.05) (Figure 3D). Neither angiotensin levels in the *L*-NAME group were influenced by ivabradine. 

After four weeks of treatment, the myocardial tissue level of Ang 1–8 was 14.98 ± 3.81 pg/g in the control group and was reduced by *L*-NAME to 7.05 ± 1.02 pg/g (53%, *p* < 0.05) (Figure 3G); other angiotensins were not detectable in the LV (low levels—below 5 pg/g).

**Figure 4:**
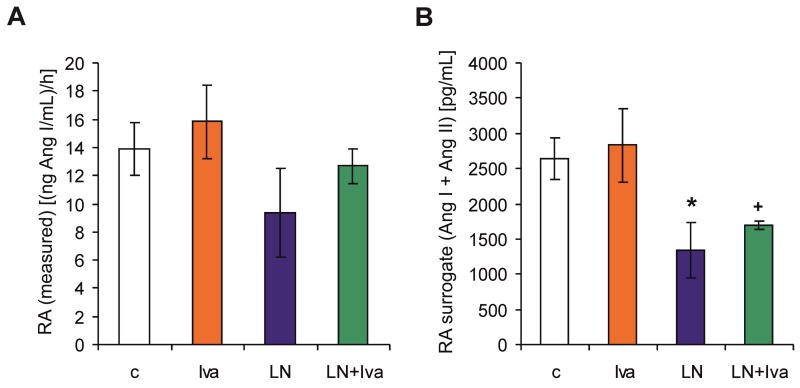
The influence of ivabradine (LN + Iva) on the measured renin activity (RA) (**A**) and surrogate renin activity (RA surrogate) (**B**) in the serum of *L*-NAME-treated rats. Wistar controls (c); *L*-NAME (LN); ivabradine (Iva); *n* = 6 per group; * *p* < 0.05 vs. c; + *p* < 0.05 vs. Iva; results are expressed as mean ± SEM.

Surrogate renin activity (RA surrogate) was 2641.30 ± 289.49 pg/mL (Ang I + Ang II) in the control group and was reduced by *L*-NAME to 1333.48 ± 397.48 pg/mL (Ang I + Ang II) (50%, *p* < 0.05) (Figure 4B). RA surrogate in the *L*-NAME group was not influenced by ivabradine. Measured renin activity (RA) was 13.88 ± 1.85 (ng Ang I/mL)/h in the control group, showing similar dynamics as the RA surrogate (reduction by 33% in the *L*-NAME group), although the changes were not significant (Figure 4A).

**Figure 5:**
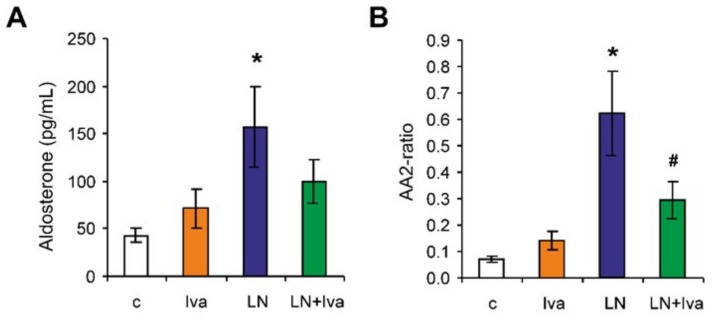
The influence of ivabradine (LN + Iva) on the serum level of aldosterone (**A**), and the aldosterone/Ang II (AA2) ratio (**B**) in *L*-NAME-treated rats. Wistar controls (c); *L*-NAME (LN); ivabradine (Iva); *n* = 6 per group; * *p* < 0.05 vs. c; # *p* < 0.05 vs. LN; results are expressed as mean ± SEM.

The serum concentration of aldosterone was 42.55 ± 7.42 pg/mL in the control group and was increased by L-NAME to 156.54 ± 42.62 pg/mL (267%, *p* < 0.05) (Figure 5A). The aldosterone/Ang II (AA2)-ratio was 0.07 ± 0.01 in the control group and was increased by L-NAME to 0.63 ± 0.16 (800%, *p* < 0.05) (Figure 5B). Ivabradine reduced the aldosterone concentration numerically (37%, ns) and decreased the AA2-ratio significantly (52%, *p* < 0.05) in the L-NAME group (Figure 5A,B).

### 2.4. Echocardiography

**Figure 6:**
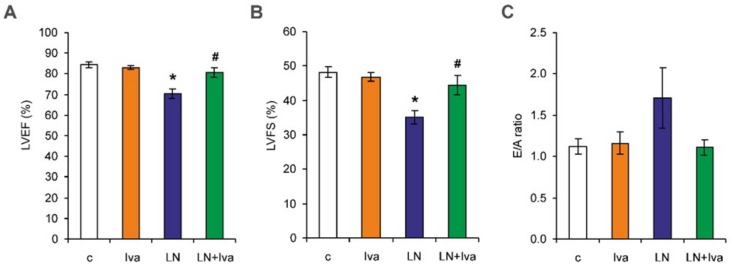
The influence of ivabradine (LN + Iva) on systolic function—left ventricular ejection fraction (LVEF) (**A**) and left ventricular fractional shortening (LVFS) (**B**), and diastolic function—the E/A ratio (**C**) of the left ventricle in *L*-NAME-treated rats. Wistar controls (c); *L*-NAME (LN); ivabradine (Iva); *n* = 6 per group; * *p* < 0.05 vs. c; # *p* < 0.05 vs. LN; results are expressed as mean ± SEM.

After four weeks of treatment, the left ventricular ejection fraction (LVEF) was 84.35 ± 1.49% in the control and was reduced to 70.33 ± 2.37% in the *L*-NAME group (17%, *p* < 0.05). Left ventricular fractional shortening (LVFS) was 48.19 ± 1.61% in the control and was reduced to 35.11 ± 1.88% in the *L*-NAME group (27%, *p* < 0.05). In the *L*-NAME group, ivabradine increased both LVEF and LVFS (15% and 27%, respectively, *p* < 0.05 for both) (Figure 6A,B). The diastolic transmitral peak early filling velocity (E velocity)/diastolic transmitral peak late filling velocity (A velocity) ratio (E/A ratio) was 1.12 ± 0.09 in the control group and was numerically increased to 1.71 ± 0.37 in the *L*-NAME group (53%, ns). In the *L*-NAME group, ivabradine numerically decreased the E/A ratio by 35% (ns) (Figure 6C).

## 3. Discussion

*L*-NAME administration increased the systolic blood pressure and left ventricular weight, enhanced the hydroxyproline concentration and content in the LV, and deteriorated the systolic and diastolic left ventricular function. Ivabradine reduced HR and SBP, improved the LV systolic and diastolic functions, and slightly reduced fibrosis. The serum equilibrium concentrations of Ang 1–8 (Ang II), Ang 1–5, Ang 1–7, Ang 1–10, Ang 2–8 (Ang III), and Ang 3–8 (Ang IV) were decreased in the *L*-NAME group and ivabradine did not modify them. These results were in line with renin activity. The concentration of the tissue Ang II in the LV was reduced in the *L*-NAME group and ivabradine did not modify it. The serum concentration of aldosterone and the AA2-ratio were enhanced by *L*-NAME and ivabradine reduced these alterations. The protective effect of ivabradine might have been associated with the reduction of the aldosterone level.

Chronic *L*-NAME administration represents an “NO-deficient” model of hypertension. In our previous experiments, *L*-NAME, the NO synthase inhibitor, reduced the activity of nitric oxide synthase in the heart, kidney, aorta, and brain [10,11,12,13]. As a result of hemodynamic and neurohumoral changes, left ventricular hypertrophy and fibrosis developed [12,14,15,16]. In this experiment, ivabradine reduced HR in both the control and *L*-NAME groups. Moreover, in *L*-NAME-hypertension, ivabradine reduced the systolic blood pressure; this result is in agreement with the previously described SBP reduction in stress-induced hypertension [17]. There are several plausible explanations for this finding. First, ivabradine improved the endothelial function in ApoE knockout mice [5,7], and increased the expression of endothelial nitric oxide synthase and exerted an anti-inflammatory effect in the aorta of hypercholesteraemic mice [18], thus preserving endothelium-dependent relaxation. Since in *L*-NAME-hypertension, deficient NO production is the principle mechanism behind hypertension development [19], improvement of the endothelial vasodilative function may result in SBP reduction. Second, ivabradine was shown to reduce the intimal hyperplasia of the aorta in hypercholesteraemic rabbits [20], reduce hypertrophy of the thoracic aorta in spontaneously hypertensive rats [9], and improve aortic compliance in ApoE-deficient mice [21], thus potentially reducing afterload. Furthermore, the reduction of the aldosterone level by ivabradine in our experiment might have contributed to SBP reduction through the diminution of circulating volume.

In this experiment, *L*-NAME administration induced LVH and fibrosis of the LV, and deteriorated systolic and diastolic LV function; ivabradine prevented these functional disturbances. The data from the literature regarding the effect of ivabradine on heart remodeling and function are quite non-homogeneous. Ivabradine reduced perivascular fibrosis in the surviving myocardium of infarcted rats [22], and reduced fibrosis and improved diastolic function in cholesterol-treated rabbits [23]. In Ang II-induced hypertension, ivabradine led to an improvement in the systolic and diastolic function, along with a reduction of cardiac hypertrophy and fibrosis [8]. Acute administration of ivabradine to chronic angiotensin II-induced hypertension improved both contraction and relaxation [24], and chronic ivabradine treatment prevented thyroid hormone-induced heart dysfunction without influencing LV hypertrophy or fibrosis [25]. On the other hand, ivabradine deteriorated the signs of HF and increased LVH and fibrosis in pressure overload-induced HF [26]. Although direct effects, i.e., on ROS generation or mitochondrial function, such as previously reported in isolated cardiomyocytes [3], cannot be ruled out, the prevention of LV dysfunction by ivabradine in our experiment might have been related to the bradycardic effect of ivabradine. A reduction of the heart rate is associated with an increased duration of diastole, supposedly resulting in improved coronary flow and energetic state of the myocardium. Although ivabradine is considered to be a direct and selective blocker of the pacemaker current (funny current, If) in the sinoatrial node [27], its possible relationship to the sympathetic system and angiotensin-aldosterone systems has yet to be fully elucidated. On the one hand, it was shown in non-diseased rats that ivabradine directly reduces HR without the modulation of HR spectral parameters, cardiovascular reflexes, or the tonic sympathovagal index, thus the HR reducing effect was not associated with the modulation of cardiovascular autonomic control [1]. However, others have found that ivabradine-induced bradycardia was related to increased sympathetic system activity resulting from baroreceptor unloading [28]. Similarly, the data with respect to ivabradine interaction with the renin-angiotensin-aldosterone system are limited. In the model of ApoE knockout mice, ivabradine reduced protein and the mRNA expression of the angiotensin II type 1 receptor (AT1 receptor) in the aorta [21], and reduced the serum Ang II level [23]. In our experiment, serum angiotensin II and its potentially beneficial downstream products (Ang 1–5, Ang 2–8, and Ang 3–8), in addition to the tissue Ang II level in the LV, were reduced in *L*-NAME rats and remained low despite the reduction of SBP by ivabradine. These results were in agreement with alterations of renin activity. It has been previously shown in our [29] and other laboratories [30,31] that it is not Ang II, but aldosterone, which is the principle player in *L*-NAME-induced hypertension and consequent peripheral organ damage, such as renal injury, independently of its systemic hemodynamic effects [31]. In this experiment, ivabradine reduced the aldosterone level (by 37%) and AA2-ratio (by 52%). A number of findings in the literature have shown that the mineralocorticoid receptor activation plays a principle role in the development and maintenance of fibrotic remodeling of the LV during hemodynamic overload [32] or heart failure [33]. It was previously shown in our laboratory that aldosterone receptor inhibition by spironolactone reduced hypertension and LV remodeling in *L*-NAME-hypertensive rats [13]. In our experiment, fibrosis of the LV was not reduced by ivabradine. However, diastolic dysfunction is a complex issue dependent on a number of different factors. Disturbed Ca^2+^ homeostasis with a delayed Ca^2+^ extrusion from the cytoplasm [34] or increased myofilament Ca^2+^ sensitivity [35] may result in abnormalities in both active relaxation and passive stiffness. Moreover, increased oxidative stress leading to reduced nitric oxide bioavailability may also result in increased cardiac stiffness [36]. Ivabradine has been shown to have antioxidant properties and to improve mitochondrial function and NO bioavailability [5,7,18]. Some of these pleiotropic actions may participate in the improvement of diastolic function by ivabradine. Aldosterone can influence the myocardium not only via its pro-proliferative effect, but also via enhancement of the oxidative burden, mineral level disorders or deteriorated endothelial function with reduced NO production [37,38]. Thus, the reduction of the aldosterone level via ivabradine could improve NO availability, and attenuate the oxidative burden, which might have contributed to the improvement of the left ventricular function. The fact that the reduction of the aldosterone concentration does not result in the attenuation of LV fibrosis may be associated with the complex regulation of fibrosis. Reduced aldosterone levels do not exclude the possible activation of the mineralocorticoid receptor by cortisol [32], which can be prevented by mineralocorticoid receptor blockade, but not aldosterone synthase inhibition [39]. Moreover, besides the RAAS, the level of endothelin, atrial natriuretic peptide, leptin, or various cytokines should be considered. One should take into account that although the aldosterone/Ang II ratio was reduced by ivabradine, the aldosterone concentration was decreased non-significantly. It seems justified to presume that if a greater number of animals were investigated for RAAS, a reduction of the aldosterone level would reach a significant difference. Nevertheless, one should interpret the potential impact of the ivabradine-induced aldosterone modification carefully.

The question of what pathomechanism stands behind ivabradine-induced reduction of the aldosterone level emerges. The secretion of aldosterone is a complex issue. Besides the well-established regulatory role of angiotensin II, aldosterone secretion is modified by a variety of other factors, such as atrial natriuretic peptide, adrenocorticotropin, K^+^, and Mg^2+^ levels [40]. There are also data indicating that NO inhibits aldosterone secretion through a cGMP-independent mechanism [41], potentially by the direct reduction of steroidogenesis or by the downregulation of AT1 receptors in the zona glomerulosa of the adrenal cortex [42]. According to the literature, ivabradine improved NO production in ApoE knockout mice [7] and in hypercholesteraemic mice [18], and reduced the AT1 receptor in the aorta [21]. In the model of chronic *L*-NAME administration, “NO-deficiency” represents a triggering factor of hypertension and peripheral organ damage. It seems reasonable to hypothesize that in the current experiment, ivabradine might have reduced the aldosterone level via improved NO availability or the downregulation of AT1 receptors in the adrenal gland.

We conclude that ivabradine improved the left ventricular function of the hypertensive heart in *L*-NAME-induced hypertension. The protective effect of ivabradine might have been associated with the reduction of the aldosterone level and aldosterone/Ang II ratio.

## 4. Materials and Methods

### 4.1. Animals and Treatment

Male adult (three-month-old) Wistar rats (Department of Toxicology and Laboratory Animals Breeding, Dobra Voda, Slovakia) were randomly divided into four groups (10 per group): age-matched control (Wistar) rats (c), rats treated with ivabradine (10 mg/kg/day) (Iva), rats treated with *L*-NAME (40 mg/kgday) (LN), and rats treated with *L*-NAME plus ivabradine in corresponding doses (LN + Iva). *L*-NAME and ivabradine were dissolved in drinking water and their concentration was adjusted to daily water consumption to ensure the correct dosage. The natural water consumption was about 12–13 mL/100 g of the body weight of the rats. In order to ensure that all of the water with dissolved ivabradine was drunk by a particular rat, only 10 mL/100 g of water-ivabradine solution was offered. The solution was prepared as follows: 10 mg of ivabradine was dissolved in 100 mL water, while no additional substance was added. All rats were housed in individual cages at 22–24 °C and fed a regular pellet diet *ad libitum*. All experimental procedures were carried out in accordance with the Guide for the Care and Use of Laboratory Animals published by the US National Institutes of Health (NIH Publication No 8523, revised 1996). The experiment was approved by the ethical committee of the Institute of Pathophysiology, Faculty of Medicine, Comenius University, Bratislava, Slovakia (approval number: 1306/14-221; approval date: 23 April 2014).

SBP was measured each week by non-invasive tail-cuff plethysmography (Hugo-Sachs Elektronic, Freiburg, Germany). After four weeks of treatment, the rats were euthanized and their BW, heart weight, LVW, and RVW were determined, and their relative weights (LVW/BW and RVW/BW ratio) were calculated. Left ventricle samples were frozen at −80 °C and later used for the determination of hydroxyproline concentrations. Blood samples were collected from the abdominal aorta during euthanasia, centrifuged at 2000× *g* for 15 min, and the obtained serum was stored at −80 °C for subsequent angiotensin and aldosterone analysis. Ivabradine (PROCORALAN^®^) was purchased from Servier Laboratories Ltd. (Suresnes, France); the remaining chemicals were purchased from Sigma Chemical Co. (Deisenhofen, Germany).

### 4.2. Determination of Hydroxyproline

The samples from the left ventricle were treated stepwise with different buffers as described previously [43]. The soluble collagenous proteins were extracted with a 0.5 mol/L CH3COOH-pepsin buffer (pH 1.4, 24 h at 4 °C) and the remaining insoluble collagenous proteins were extracted with 1.1 mol/L NaOH (45 min at 105 °C). A hydroxyproline concentration (a marker of fibrosis) was estimated in both collagenous fractions using spectrophotometry. Oxidation of the hydrolyzed samples was started with the addition of chloramine T in acetate-citrate buffer, pH 6.0. The mixture was incubated for 20 min at room temperature and the reaction was stopped by adding 20 volume of Ehrlich’s reagent solution. After the incubation of samples at 65 °C for 15 min, the hydroxyproline concentration was measured spectrophotometrically at 550 nm [44]. The hydroxyproline content was expressed in mg per total weight of the LV.

### 4.3. The Serum Concentration of Angiotensins and Aldosterone, the Concentration of Angiotensin in the LV and Renin Activity (Measured and Surrogate)

Equilibrium angiotensin peptide concentrations and aldosterone levels were determined by mass spectrometry in the serum samples as described previously [45]. Briefly, the conditioned serum was equilibrated at 37 °C for 30 min, followed by the stabilization of equilibrium peptide levels. The stabilized samples were further spiked with stable isotope-labelled internal standards for each angiotensin metabolite (Ang I, Ang II, Ang 1–7, Ang 1–5, Ang 2–8, and Ang 3–8) and aldosterone at concentrations of 200 pg/mL and 500 pg/mL, respectively. Following C18-based solid-phase-extraction, the samples were subjected to LC-MS/MS analysis using a reversed-phase analytical column (Acquity UPLC^®^ C18, Waters Corp., Milford, MA, USA) operating in line with a XEVO TQ-S triple quadrupole mass spectrometer (Waters Corp.) in MRM mode. Internal standards were used to correct for the peptide recovery of the sample preparation procedure for each angiotensin metabolite in each sample. Angiotensin peptide concentrations were calculated considering the corresponding response factors determined in appropriate calibration curves in the original sample matrix on the condition that integrated signals exceeded a signal-to-noise ratio of 10. Six animals per group were used for the analysis of angiotensins and aldosterone. Angiotensin peptide concentrations in samples of the LV were determined by mass spectrometry as described previously [46].

For renin activity, blood was collected from rats, left to coagulate, and centrifuged. The obtained serum was diluted in an Ang I generation buffer containing ethylenediaminetetraacetic acid (5 µM), Z-Pro-Prolinal (20 µM), 4-(2-Aminoethyl)benzenesulfonyl fluoride hydrochloride (1 mM), and aminopeptidase inhibitor (10 µM) in phosphate-buffered saline (Dulbecco’s PBS, pH 7.4). Mixtures were split into two aliquots, one of which was mixed with additional aliskiren (10 µM) as the baseline control. Following incubation at 37 °C, the mixtures were stabilized and further processed for Ang I quantification as all angiotensins in equilibrium analysis. Serum renin activity (RA) was calculated by subtracting Ang I formation in the aliquot with aliskiren from the aliquot without aliskiren. The chemicals were purchased from Sigma-Aldrich (Munich, Germany).

RA-surrogate was calculated as the sum of Ang I and Ang II, since it has been shown that the sum of Ang I and Ang II, obtained from the equilibrium analysis, tightly correlated with the measured plasma renin activity, independent of applied treatment or species used [47]. 

### 4.4. Echocardiography

After four weeks of treatment, transthoracic echocardiography was performed on six animals per group by an experienced echocardiographer blinded to the group identity. A 14-MHz matrix probe (M12L) coupled with a GE Medical Vivid 7 Dimension System (GE Medical Systems CZ Ltd., Prague, Czech Republic) was used. Anesthesia was maintained by applying a 2.5% inspiratory concentration of isoflurane at a flow rate of 2 L/min through a sealed nose cone during spontaneous breathing. The rat was placed in the supine position on a warming pad (38 °C) and the thoracic wall was shaved. The heart rate and body temperature were monitored continuously. To assess left ventricular systolic function, the LV end-systolic and end-diastolic internal diameters were measured by the leading-edge method from the anatomical M-mode in long axis view. Left ventricular fractional shortening (LVFS) was calculated as described previously [45]. Left ventricular ejection fraction (LVEF) was determined by the Teichholz formula. To asses left ventricular diastolic function, the diastolic transmitral peak early filling velocity (E velocity) and the diastolic transmitral peak late filling velocity (A velocity) were measured, and their ratio (E/A) was calculated from the two-dimensionally guided Doppler spectra of mitral inflow in apical four-chamber view. All measurements were averaged over three consecutive cardiac cycles [48,49].

### 4.5. Statistical Analysis

The statistical analysis was performend using NCSS 11 Statistical Software 2016 (NCCS, LCC. Kaysville, UT, USA) and TIBCO, Statistica (data analysis software system) version 13 Software Inc. 2017 (TIBCO Software Inc., Palo Alto, CA, USA). The results are expressed as means ± SEM. All the data were tested for normality. One-way repeated measures analysis of variances with a post-hoc Fisher’s LSD test was used to evaluate differences in systolic blood pressure and heart rate. The other parameters were analyzed by one-way analysis of variance with the post-hoc Fisher‘s LSD test. The level of significance was *p* < 0.05.

## 5. Limitations

It is a challenge to reveal the mechanisms standing behind the effect of ivabradine on the RAAS and the heart function. The hemodynamic changes might have influenced the level of RAAS. The investigation of RAAS in our experiment has indicated that the aldosterone level was increased and angiotensin II and its downstream products were reduced in *L*-NAME-hypertension. Previously, ivabradine has been shown to reduce oxidative stress and improve endothelial function [5,7] associated with a potential vasodilation of the renal artery. Thus, it might have reduced renin release and maintained the low level of Ang II, and even showed a clear trend to reduce the level of aldosterone despite blood pressure reduction. Hemodynamic changes might have influenced the cardiac function either directly (via improving the energetic state of myocardium) or indirectly (through modification of neurohumoral cascades). Besides the renin-angiotensin-aldosterone system, the vegetative nervous system, nitric oxide pathway, and endothelin or atrial natriuretic peptide levels should also be investigated. In addition, the determination of AT1 receptor number in the adrenal cortex and peripheral vasculature may be of value. A rational way to distinguish between direct hemodynamic effects and the concomitant neurohumoral activation may be achieved by the equilibration of BP in all investigated groups by a substance with a declared pure vasodilatative action, such as hydralazine or a calcium channel blocker. However, such investigations were beyond the scope and possibilities of this work.

## Abbreviations

HR	Heart rate
LV	Left ventricle
*L*-NAME	N^G^-nitro-*L*-arginine methyl ester
Ang	Angiotensin
(AA2)-ratio	Aldosterone/angiotensin II ratio
HF	Heart failure
LVH	Left ventricular hypertrophy
Iva	Ivabradine group
LN	N^G^-nitro-*L*-arginine methyl ester group
c	Control group
LN+Iva	N^G^-nitro-*L*-arginine methyl ester plus ivabradine group
BW	Body weight
LVW	Left ventricular weight
RVW	Right ventricular weight
LVFS	Left ventricular fractional shortening
LVEF	Left ventricular ejection fraction
E velocity	Diastolic transmitral peak early filling velocity
A velocity	Diastolic transmitral peak late filling velocity
If	Funny current
RAAS	Renin-angiotensin-aldosterone system
SBP	Systolic blood pressure
NADPH	Nicotinamide adenine dinucleotide phosphate
